# Communication is key: an innovative multidisciplinary approach to communication of regional antimicrobial resistance surveillance data to hospital microbiologists in North East England

**DOI:** 10.1186/2047-2994-4-S1-P166

**Published:** 2015-06-16

**Authors:** SJ Howard

**Affiliations:** 1 North East Health Protection Team, Public Health England, Newcastle upon Tyne, UK

## Introduction

Antimicrobial resistance (AMR) must be urgently tackled [1]. Strategies to tackle AMR must include using high quality surveillance data to rapidly modify policy and practice. Yet, in the UK and elsewhere, AMR surveillance is conducted by specialists located in organisations separate from hospital practitioners. This leads to communication challenges, including delays, difficulty in communicating technical limitations around interpretation of data, and a lack of clinical interpretation of surveillance data.

## Objectives

This project aimed to improve the quality, speed and clinical relevance of communication between hospital microbiologists and surveillance specialists with regard to AMR surveillance data.

## Methods

A multidisciplinary virtual working group was convened, including hospital microbiologists, the regional microbiologist, a consultant epidemiologist, and a senior information officer, led by a public health specialty registrar. This group developed a short quarterly regional AMR report format, showing and statistically analysing trends, and giving a commentary on the data limitations and clinical significance of the data. The report is thematic, responding to queries and concerns of front-line microbiologists. Discussion of this report at an existing regional microbiology group was encouraged.

## Results

Feedback regarding the “North East AMR Quarterly Report” to date has been universally positive. The level of engagement with this report, as measured by communication with the surveillance team, has been greater than for previous regional AMR surveillance reports, and it has been praised for its clear focus on clinical relevance in particular.

## Conclusion

Surveillance is a key tool for tackling AMR. Surveillance data can be a useful tool to influence front-line practice, but to be most effective, it must be interpreted rather than presented in raw formats whose limitations and clinical relevance may not be clear. A multidisciplinary approach to creating data-driven reports was of particular value in this case, and may be worthy of consideration elsewhere.

## Disclosure of interest

None declared.
